# Feasibility of Reflectance Confocal Microscopy Monitoring in Oily, Acne-Prone Facial Skin Treated with a Topical Combination of Alpha and Beta-Hydroxy Acids, Anti-Inflammatory Molecules, and Herculane Thermal Water: A Blinded, One-Month Study

**DOI:** 10.3390/life12121973

**Published:** 2022-11-25

**Authors:** Mihai Lupu, Ana Maria Malciu, Vlad Mihai Voiculescu

**Affiliations:** 1 Department of Dermatology, MEDAS Medical Center, 030442 Bucharest, Romania; 2 Department of Dermatology, “Carol Davila” University of Medicine and Pharmacy, 050474 Bucharest, Romania; 3 Department of Dermatology and Allergology, Elias Emergency University Hospital, 011461 Bucharest, Romania

**Keywords:** acne-prone skin, oily skin, reflectance confocal microscopy, non-invasive skin imaging, acne vulgaris

## Abstract

Oily, acne-prone skin is a common skin type which may be monitored in vivo using reflectance confocal microscopy (RCM). The aim of the study was to assess the feasibility of RCM in evaluating the effectiveness of a topical combination of alpha- and beta-hydroxy acids, anti-inflammatory and antibacterial molecules, and Herculane thermal water on acne-prone skin. Thirty-five subjects with oily, acne-prone skin were prescribed topical combination products and were evaluated by clinical, Wood’s lamp, and RCM imaging at baseline and after 28 days. At 28 days, the RCM-evaluated number of dilated infundibula, infundibula filled with keratotic material, and infundibula with thickened bright borders, as well as the density of the inflammatory infiltrate, were significantly decreased. Wood’s light images at 28 days showed a significantly reduced number of *C. acnes*-colonized infundibula, and both the median area and the intensity of the red-orange fluorescence were decreased. The reduction in the clinical score was concurrent with the improvement in the RCM parameters, suggesting that this non-invasive imaging technique is appropriate for efficiency evaluations of topical acne treatments.

## 1. Introduction

Oily, acne-prone skin and acne vulgaris are very common conditions. They primarily affect adolescents and have characteristic clinical appearances. Altered and increased androgen-mediated sebum production, keratinocyte hyper-proliferation in the infundibulum, follicular colonization by *Cutibacterium acnes*, and inflammatory infiltrates are key components of the pathologic process in both acne-prone skin and acne, and thus represent main therapeutic targets [1,2].

Different management approaches for the treatment of acne and acne-prone skin [3] are currently available. Mild forms of acne are routinely treated with topical products, including comedolytic and anti-inflammatory agents [3]. Treatment effectiveness is usually assessed through clinical and laboratory evaluations [4,5,6,7]. Classic evaluation methods of disease evolution and treatment efficacy include questionnaires, clinical examination, and macro-photography and dermoscopy of acne lesions. Although sufficient in most cases, these methods do not evaluate the clinically imperceptible microscopic changes one would expect during the first few weeks of treatment and thus cannot appreciate the therapeutic effect until it reaches a certain threshold. Furthermore, classic evaluation methods cannot discern which pathogenic component is influenced by therapy.

Microscopic changes occurring inside infundibula and the inter-infundibular epidermis during treatment have been difficult to evaluate due to the need for a series of biopsies of cosmetic sensitive areas. However, the introduction of in vivo reflectance confocal microscopy (RCM) for skin imaging offers the unique opportunity to perform optical biopsies at quasi-histological resolution without tissue damage [8,9,10]. Moreover, due to its non-invasiveness, RCM allows repeated imaging of the same area over time [11,12]. A previous study [13] has shown that the in vivo identification of the main histopathologic aspects of acne-prone skin and the subclinical alterations present in apparently healthy skin of subjects with this phenotype, namely, microcomedos, infundibular hyper-keratinization, accumulation of keratotic and sebaceous material, and the presence of inflammatory infiltrate, is possible.

The purpose of this prospective, one-month study was to assess the feasibility of RCM in evaluating the effectiveness of a topical combination of alpha- and beta-hydroxy acids, anti-bacterial agents, anti-inflammatory molecules, and Herculane thermal water (Ivapur K cream) used in conjunction with a cleanser (Ivapur purifying cleansing gel) on oily, acne-prone skin of the face.

## 2. Materials and Methods

The study was conducted in accordance with the ethical principles of the Declaration of Helsinki and in compliance with local regulatory requirements. The study was reviewed and approved by the ethics committee (protocol no. 2329/18.03.2022).

### 2.1. Study Population

Subjects were recruited from in-office consultations in two dermatology departments in Bucharest: Panduri Medical Center and the Oncologic Dermatology and Allergology Clinic of the “Elias” University Emergency Hospital.

Subjects over the age of 12, male and female, Fitzpatrick phototypes II and III, with oily, acne-prone skin, and without any other skin conditions (aside from possibly mild vulgar acne) at the examined sites were considered eligible for inclusion.

Subjects who had been enrolled in another study in the past 30 days, presented a comorbidity which prevents them from being enrolled, presented specific skin issues (dermatitis, scarring, tattoos, sun burns) in the examined areas, pregnant or nursing women, topical or systemic corticosteroid use in the past 6 months, severe forms of acne, known allergies to any of the topical formulation ingredients, aesthetic procedures (e.g., chemical peels, lasers, etc.) undergone in the past 60 days, and/or extensive UV radiation (artificial or natural) exposure in the past 30 days were excluded from the study.

All subjects were informed both verbally and in writing about the nature and methodology of the study and signed informed consent prior to being enrolled. Patients were asked to return for a second evaluation visit and documentation after 28 days of treatment.

Due to the post-approval nature of this study, we did not perform study size calculations. Following a review of the literature, we decided to enroll a total of 40 subjects.

### 2.2. Topical Products and Their Posology

The two topical products used in this study were Ivapur K cream and Ivapur purifying cleansing gel, from Ivatherm. Ivapur K cream is a combination of, among other ingredients, lactic acid, salicylic acid, piroctone olamine, tea tree oil, bisabolol, and Herculane spring thermal water (the complete ingredient list has been made available by the manufacturer). Ivapur purifying cleansing gel contains, among other ingredients, zincidone, glycerine, and Herculane spring thermal water (the complete ingredient list has been made available by the manufacturer).

Subjects were instructed to apply the products on their face twice a day (morning and evening) for 28 consecutive days. Product application instructions were given in written format to all subjects: the face should first be cleansed with Ivapur purifying cleansing gel (applied on moist skin and then gently rubbed until foaming, then washed away with lukewarm water), dabbed dry with a clean cotton towel, and then Ivapur K cream should be applied in circular motion until absorbed into the skin.

### 2.3. Data Acquisition

#### 2.3.1. Sebum Production Quantification

Sebum production was assessed through the validated SOS (skin oiliness scale) questionnaire [14], which was filled out following subject questioning at baseline (day 0) and after 28 days. The questionnaire contains six items which, depending on the given answer, each receive from 1 to 5 points. The total score is the sum of each item’s score and thus varies between 6 and 26. A higher total score indicates a more abundant sebum production.

#### 2.3.2. Instrument and Image Acquisition (RCM and Wood Lamp)

Clinical and RCM imaging were acquired at baseline and after 28 days to evaluate the effectiveness of the applied products.

Clinical digital photographs of the entire face and of the target area were taken by means of a digital camera (Canon 1100D, Canon, Japan). Dermoscopic images were then obtained through the VivaCam (Caliber ID, Rochester, NY, USA) dermoscope.

All reflectance confocal microscopy images were acquired in the Panduri Medical Center, Bucharest, using a commercially available confocal microscope (VivaScope 1500 Gen 4, Caliber ID, Rochester, NY, USA). A target area of 4 × 4 mm on the medial line of the forehead, one centimeter above the eyebrow line was selected for imaging. Three individual 4 × 4 mm mosaics (stitched horizontal optical slices) in 30 µm depth increments starting at the stratum corneum were obtained. Consecutively, individual stacks (serial horizontal optical slices varying in depth similar to a punch biopsy) of infundibula or acne lesions (if present) down to a depth of 200–250 µm were acquired.

Using the same digital camera (Canon 1100D, Canon, Japan), clinical images under Wood’s light were acquired at baseline and after 28 days. The Wood lamp used in this study was the Dermalight 80R Woodlight (Dr. Hönle Medizintechnik GmbH, Germania), which emits ultraviolet radiation with a wavelength of approximately 365 nm. Three incidences were captured: frontal plane, right profile, and left profile.

#### 2.3.3. Measurements

All evaluations were performed in by researchers blinded to the clinical images and data after hiding and randomizing the patient’s data and visit number.

**Figure 1:**
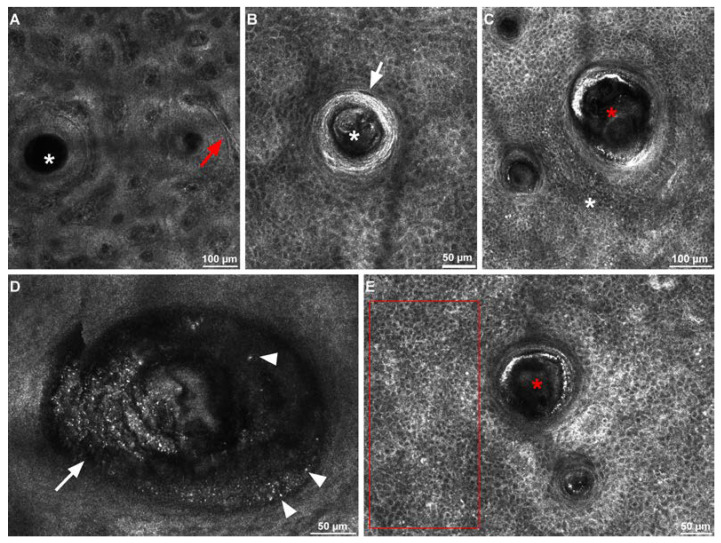
(**A**) RCM image of regular follicular infundibula (white asterisk) at the level of the dermal-epidermal junction and interfollicular dilated vessels (red arrow). (**B**) RCM image of an infundibulum (white asterisk) circumscribed by a thickened, hyper-reflective border surrounded (white arrow) by normal appearing epidermis. (**C**) RCM image of a dilated infundibulum (red asterisk) with hyper-reflective border and mild peri-follicular inflammatory infiltrate (white asterisk). (**D**) RCM image showing a dilated infundibulum containing amorphous reflective material (white arrow) and hyper-reflective particles—dots (white arrowheads). (**E**) RCM image showing an infundibulum (red asterisk) presenting thickened, bright border and hyper-reflective particles scattered in the proximity (red rectangle), representing moderate inflammatory infiltrate.

Reflectance confocal microscopy images were evaluated by an experienced RCM user (ML) through Image J (National Institutes of Health, MD, USA), an open-source image analysis software package. RCM evaluations were focused on (a) the count of hair follicles presenting normal infundibula (Figure 1A); (b) the number of infundibula with abnormal keratinization described as a hyper-reflective border (Figure 1B); (c) the number of dilated infundibula/comedos (>150 µm) corresponding to round-oval structures presenting a hyper-reflective border (Figure 1C); (d) the number of follicles with intra-infundibular hyperkeratotic contents corresponding to amorphous material with mid to high reflectivity (Figure 1D); (e) the mean infundibular diameter (manually measured from the three most dilated infundibula in the target area); (f) the number of infundibula containing hyper-reflective particles named dots (Figure 1D);(g) the density (mild, moderate, dense) of the inflammatory infiltrate corresponding to bright particles in the inter-follicular epidermis (Figure 1E); and (h) inter-follicular vascular changes described as dark cannalicular spaces which on RCM real-time images contain rapidly moving, bright particles corresponding to blood cells (Figure 1A).

**Figure 2:**
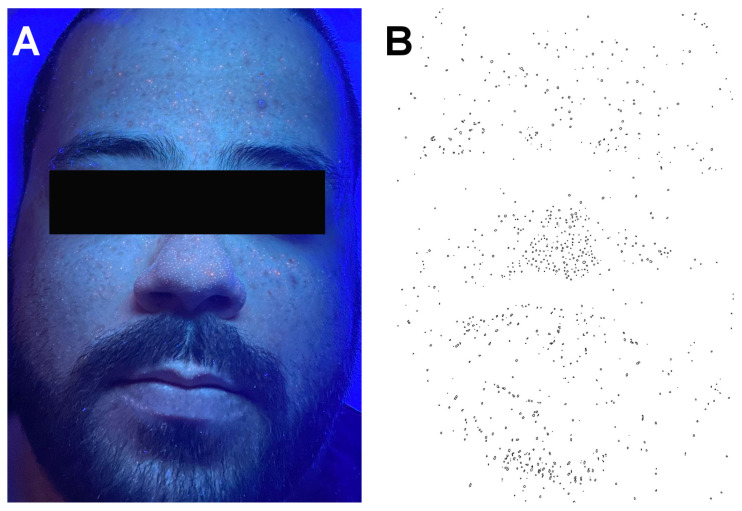
Clinical image taken in Wood’s light. (**A**) The bright red-orange dots represent infundibula colonized by *Cutibacterium acnes*. (**B**) The image in panel A after being put through the segmenting algorithm; black dots represent infundibula colonized by *C. acnes*.

Wood’s light images were analyzed in Image J (National Institutes of Health, Bethesda, MD, USA) through a trainable segmentation algorithm. After segmentation, data regarding (a) the total number of follicular infundibula colonized by *Cutibacterium acnes* (corresponding to red-orange fluorescence) (Figure 2), (b) the total red-orange fluorescence area (expressed as percentage of the total facial area), and (c) mean red-orange fluorescence intensity (manually measured from the three most bright spots within the study area) were retrieved.

### 2.4. Statistics

Continuous variables such as SOS score and RCM parameters (number of regular follicles, number of infundibula with thickened bright borders, number of follicles with dilated infundibula, number of comedos, number of follicles with intra-infundibular bright dots) were expressed as mean and standard deviation. Student’s *T*-test was used for mean comparisons between baseline and day 28.

Other variables and ordinal variables were expressed as median and IQR (inter-quartile range). Wilcoxon Rank test was used to analyze any parameter fluctuations in the two stages of the study. Confidence intervals were set at 95%, and a *p* value < 0.05 was considered significant. All statistical analyses were performed using SPSS v20 (IBM^®^, Armonk, NY, USA) software package.

## 3. Results

A total number of 40 subjects were enrolled, yet only 35 completed the study (17 males and 18 females) and were included in the final analysis. Five subjects (all female) dropped out of the study by failing to attend the final visit at 28 days.

Subjects had a mean age of 19.17 ± 6.37 years old (range 12–35), and only seven of them were smokers. No product-related side effects were reported during this study.

**Figure 3:**
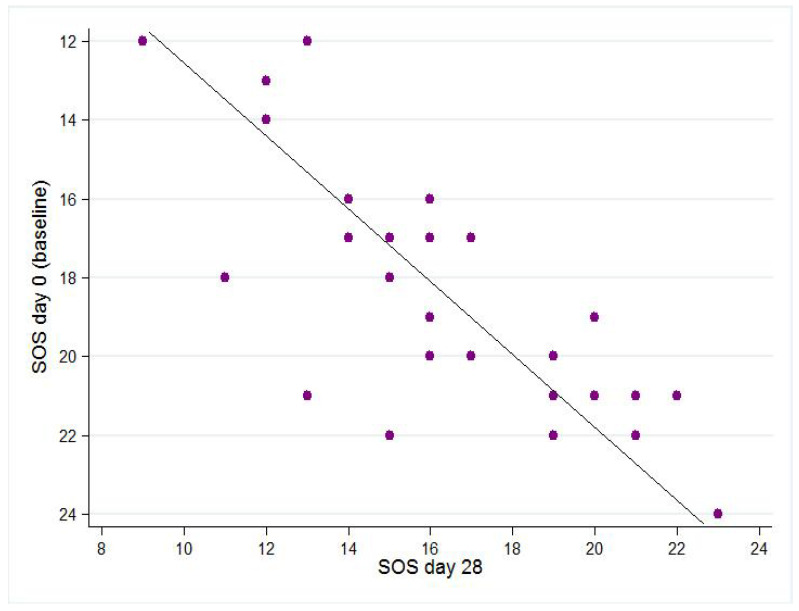
SOS (skin oiliness scale) score at baseline compared to day 28.

The average SOS score showed a significant decrease of 2.05 points between baseline and day 28 (t (1.33; 2.77) = 5.79, *p* < 0.001) (Figure 3).

At baseline, RCM evaluations showed an average of 68.49 ± 21.97 hair follicles detectable in the 4 × 4 mm areas, of which 60.11 ± 20.76 were regular, 8.37 presented infundibula with thickened bright borders corresponding to hyperkeratosis, 7.71 had dilated infundibula, 5.46 presented intra-infundibular amorphous, reflective material, representing comedos, and 3.17 presented with hyper-reflective particles representing dots. The median infundibular diameter at baseline, as measured on RCM images, was 172.25 (IQR 50.73) μm. On day zero, the inflammatory infiltrate was mostly mild (20/35), and only two subjects (2/35) showed inter-follicular hyper-vascularity.

**Figure 4:**
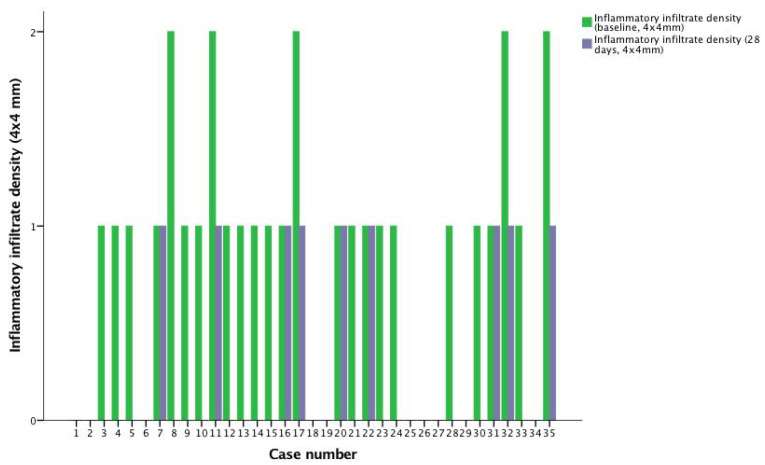
Inflammatory infiltrate density in each case on baseline and on day 28.

**Figure 5:**
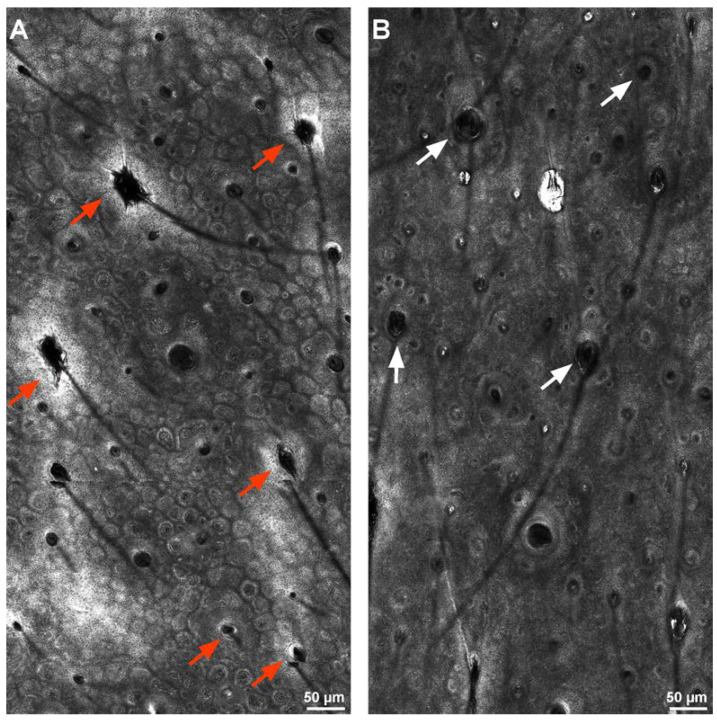
Reflectance confocal microscopy (RCM) images of a 24-year-old woman’s skin. (**A**) RCM image at baseline, before topical product use, showing multiple infundibula with hyper-reflective borders (red arrows). (**B**) RCM image after 28 days of topical product use showing predominantly regular infundibula (white arrows) without hyper-reflective borders.

At day 28, the average number of infundibula with thickened bright borders decreased from 8.37 to 5.08 (*p* < 0.001), while the number of regular follicles increased from an average of 60.11 to 63.4 (*p* < 0.001). The average number of follicles with dilated infundibula decreased from 7.71 to 6.57 (*p* = 0.005), and so did the number of comedos (from 5.45 to 3.71, *p* < 0.001) and intra-infundibular bright dots (from 3.17 to 1.94, *p* < 0.001). Measurements of the infundibular diameter on RCM images taken at day 28 showed an average reduction of 14.76 μm, which was not statistically significant (*p* = 0.241). At the same time, a Wilcoxon signed-rank test showed that at 28 days, the topical treatment course elicited a statistically significant decrease in inflammatory infiltrate density (*Z* = −4.379, *p* < 0.001) (Figure 4). The number of subjects presenting with inter-follicular vascular changes was also reduced (from two subjects to only one), although without reaching significance (*p* = 0.317). RCM parameter changes have been summarized in Table 1. Figure 5 shows RCM images of the study area of a patient at baseline and at 28 days. Note the significantly reduced number of bright-bordered infundibula and infundibula containing amorphous material at 28 days.

Baseline Wood’s light image analysis showed a median number of 466 follicles colonized by *Cutibacterium acnes* in the entire facial area. The median red-orange fluorescence area was 0.278 (IQR 0.31), and the median fluorescence intensity was 197.1 (IQR 32.08).

**Figure 6:**
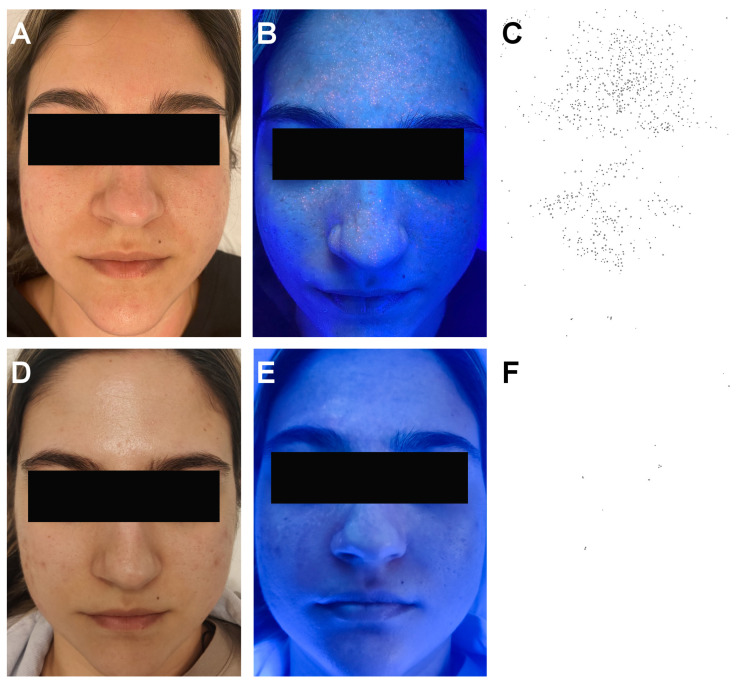
Clinical, Wood’s light, and post-segmentation images. (**A**) Clinical image of the patient at baseline. (**B**) Wood’s light image at baseline showing numerous red-orange fluorescent dots (*C. acnes*-colonized infundibula) covering the forehead, nose, and cheeks. (**C**) Post-segmentation image showing the colonized facial area in black. (**D**) Clinical image of the patient after 28 days of topical product use. (**E**) Wood’s light image after 28 days of topical product use—note the significant reduction in the number of red-orange fluorescent dots. (**F**) Post-segmentation image after 28 days of topical product use showing a significantly reduced colonized area in black.

At the end of the study on day 28, the number of follicles colonized by *Cutibacterium acnes* was significantly reduced by an average of 72 (*p* = 0.003), and so were the average red-orange fluorescence area (from 0.278 to 0.227, *p* = 0.011) and intensity (from 197.1 to 175.25, *p* = 0.001). Parameter evolution has been summarized in Table 2. Figure 6 depicts clinical images under Wood’s lamp and the corresponding segmented images at baseline and after 28 days. Note the reduced number of *C. acnes*-colonized follicles at 28 days.

## 4. Discussion

In this study, RCM is employed for the evaluation of the effect of topical products Ivapur K cream and Ivapur purifying cleansing gel on oily, acne-prone skin. The products were applied twice daily, morning and evening, on the entire face. Being non-invasive, RCM gives us the possibility to repeat the imaging over time on the same areas, thus permitting the identification of the microscopic changes which occur in the hair follicles and the surrounding skin with topical product use.

Sebum production, the main characteristic of oily skin, is quantified using a validated, six-item questionnaire [14]. There was a significant two-point decrease in the average SOS score at day 28, thus confirming the seboregulatory properties of the two topical products. Overall, the reduction in the SOS clinical score (significant after 28 days) was concurrent with the improvement in RCM parameters associated with oily, acne-prone skin.

The study of acne-prone skin by means of RCM using the VivaScope 1500 allows for the identification and quantitation of pilo-sebaceous infundibular changes occurring in oily, acne-prone skin. Altered keratinization in the shape of thick, hyper-reflective infundibular borders has already been identified as a trait of apparently normal skin in acne-prone patients [13]. Infundibula hyper-keratinization may be regarded as the early event leading to microcomedo genesis. Cunliffe et al. argue that hyper-proliferation of ductal keratinocytes and turnover imbalance induced by hormonal factors lead to the accumulation of cells causing ductal obstruction, thus initiating the events that induce comedo development [15].

At the end of the 28-day study period, the reduction in the RCM number of infundibula with thick, hyper-reflective borders, dilated infundibula, and comedos was counterbalanced by the increase in the number of regular infundibula with normal borders. In particular, the ratio between the number of regular infundibula and the number of hyper-reflective borders infundibula significantly increased (from 7.18 at baseline to 12.48 at the end of the study). These data suggest that the effect of topical products containing exfoliating agents such as alpha- and beta-hydroxy acids, which slowly act on the hyperkeratotic component with a visible effect on the infundibula structure after 28 days, can be evaluated through RCM. Achieving normalization of the infundibular structures is directly tied to the prevention of new lesion formation and maintaining long-term efficacy through topical product application, which could also prove beneficial as adjuvant therapy during systemic treatments.

The significant reduction at 28 days in the average number of *Cutibacterium acnes*-colonized follicles, red-orange fluorescence area and intensity is in line with the reported efficacy of tea tree oil as an antibacterial agent [16].

In all, RCM proved a feasible method for evaluating the microscopic changes in acne-prone skin during treatment. Moreover, the treatment protocol employed in this study was effective in curbing the key components of oily, acne-prone skin and acne vulgaris over the course of the 28-day study period. The in vivo study of topical product dynamics improves knowledge on the microscopic changes occurring in the skin during therapy, thus providing more information on the correct approach for treatment optimization. There is a need for further larger comparative studies to confirm these initial data. We consider reflectance confocal microscopy imaging to represent a unique approach for the objective assessment of topical product efficacy on oily, acne-prone skin.

## Figures and Tables

**Table 1:** RCM parameters at baseline and at the end of the study period on day 28.

	Baseline Mean (SD)	Day 28 Mean (SD)	*p*
Regular follicles and infundibula	60.11 (20.76)	63.4 (22.27)	<0.001
Infundibula with bright, thickened borders	8.37 (6.18)	5.08 (3.63)	<0.001
Dilated infundibula (>150 μm)	7.71 (3.95)	6.57 (3.64)	0.005
Infundibular diameter (median, μm)	172.25	157.49	0.241
Infundibula containing amorphous material (comedos)	5.45 (2.28)	3.71 (2.03)	<0.001
Intra-infundibular dots	3.17 (2.1)	1.94 (1.95)	<0.001
Inter-follicular vascular changes	2	1	0.317

RCM, reflectance confocal microscopy; SD, standard deviation.

**Table 2:** Wood’s light parameters at baseline and at the end of the study period.

	Baseline	Day 28	*p*
Follicles colonized by *C. acnes* (median)	466	394	0.003
Area of red-orange fluorescence (median, IQR)	0.278 (0.31)	0.227 (0.31)	0.011
Red-orange fluorescence intensity (median, IQR)	197.1 (32.08)	175.25 (60.33)	0.001

IQR, interquartile range.

## Data Availability

Not applicable.
